# Impact of embryo size on apoptosis in *C. elegans*

**DOI:** 10.17912/micropub.biology.001608

**Published:** 2025-05-19

**Authors:** Madiha Javeed Ghani, Jens Van Eeckhoven, Barbara Conradt

**Affiliations:** 1 Cell and Developmental Biology, University College London, London, England, United Kingdom

## Abstract

During *C. elegans* development 131 somatic cells reproducibly undergo programmed cell death. Many of these 131 cells ‘programmed to die' are the smaller daughter of a neuroblast that divides asymmetrically and die through apoptosis. To determine whether cell size impacts the ability of cells programmed to die to undergo apoptosis, we increased or decreased embryo size by RNA interference-mediated knock-down of the genes *C27D9.1* or *ima-3* , respectively. We found that in apoptosis-compromised genetic backgrounds, *C27D9.1* ( *RNAi* ) enhances and *ima-3* ( *RNAi* ) partially suppresses inappropriate survival of cells programmed to die. This supports the notion that in *C. elegans* embryos, an increase in cell size compromises and a decrease in cell size promotes the ability of cells programmed to die to undergo apoptosis.

**Figure 1. RNAi-mediated embryo size alterations and impact on the apoptotic death of the NSM sister cell:**
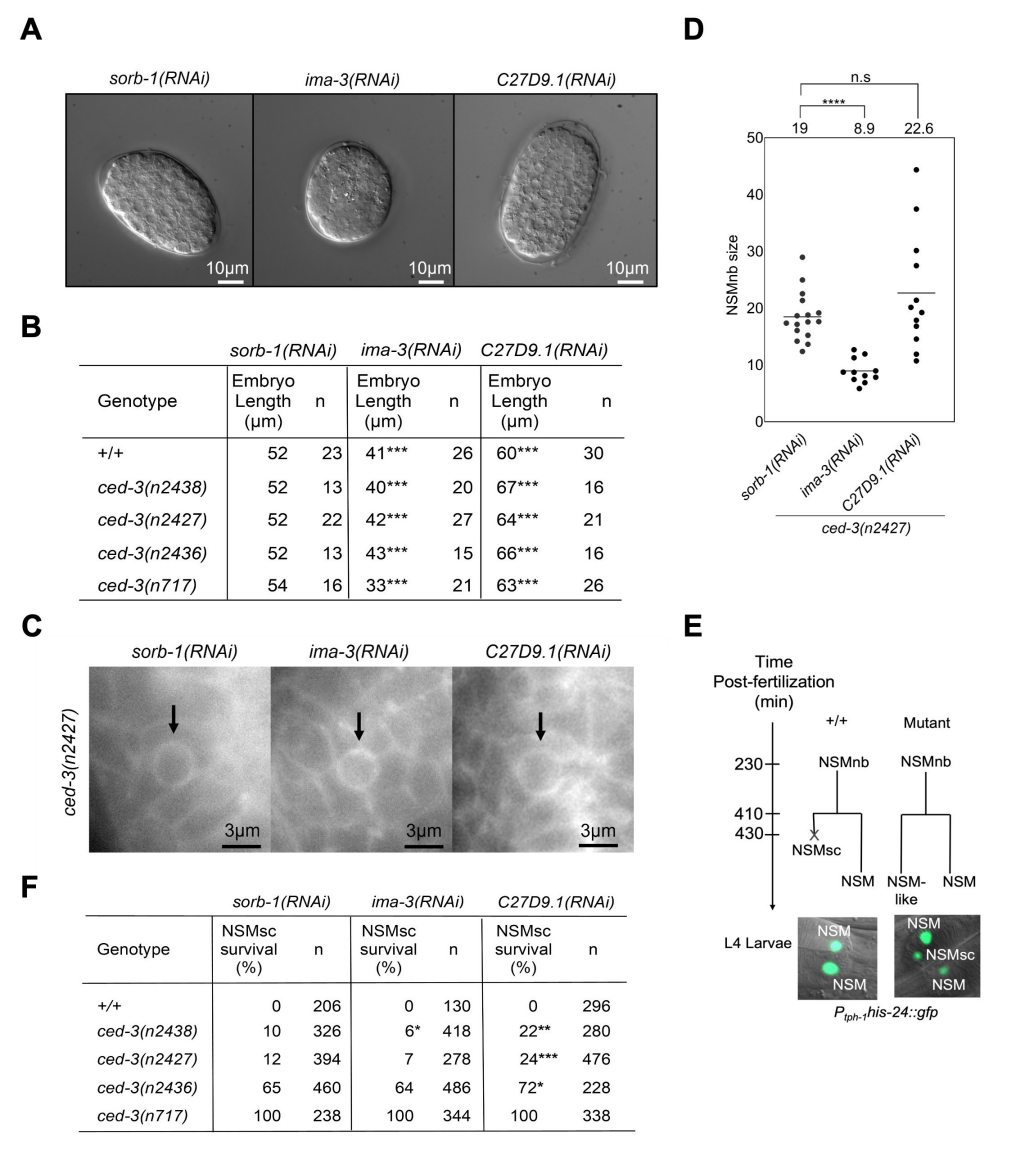
(A) RNAi-mediated embryo size alterations. DIC images of representative embryos following *sorb-1 (RNAi)* , *ima-3 (RNAi)* or *C27D9.1 (RNAi)* are shown. Scale bars: 10μm. All embryos were analysed at a similar stage of embryonic development (about 220-cell stage). (B) Average embryo length after RNAi-mediated embryo size alterations. For statistical comparison, the lengths of *ima-3 (RNAi)* or *C27D9.1 (RNAi)* embryos were compared to the lengths of *sorb-1 (RNAi)* embryos *.* Statistical significance was determined by least square means method. *p* <0.001(***). (C) RNAi-mediated NSM neuroblast (NSMnb) size alterations. Fluorescence images of representative NSMnb following *sorb-1 (RNAi)* , *ima-3 (RNAi)* or *C27D9.1 (RNAi)* are shown. The *P _pie-1_ mCherry::ph ^PLCΔ^* reporter ( *ltIs44* ) was used to visualize cell boundaries. Scale bars: 3μm. (D) Average NSMnb size measurements after RNAi-mediated embryo size alterations. Each dot represents the estimated size of one NSMnb. The average size for each RNAi treatment is shown above the graph. Statistical significance was determined by Mann-Whitney U-tests. *p* <0.0001(****). (E) Schematics of the NSM lineage and the NSMsc survival assay. In wild type ( *+/+* ), the NSMsc dies and its larger sister differentiates into the NSM neuron, which can be visualized in the anterior pharynx of L3/L4 larvae using the *P _tph-1_ his-24 ::gfp* reporter ( *bcIs66* ). There are two bilaterally symmetric NSM lineages. For this reason, in *+/+* there are two GFP-positive cells in the anterior pharynx. If the NSMsc inappropriately survives ('Mutant'), three to four GFP-positive cells are observed. (F) Average NSMsc survival (%) after RNAi-mediated embryo size alterations. NSM survival for each RNAi treatment was scored in a wild-type ( *+/+* ) background and in the background of different *ced-3* loss-of-function mutations ( *n2438 , n2427 , n2436 , n717* ). All strains used were homozygous for the *P _tph-1_ his-24 ::gfp* reporter ( *bcIs66* ). For statistical comparison, NSMsc survival in *ima-3 (RNAi)* or *C27D9.1 (RNAi)* was compared to NSMsc survival in *sorb-1 (RNAi)* for each genotype. One sided Fisher's exact tests were used to determine statistical significance. *p* <0.05 (*), *p* <0.01(**) and *p* <0.001(***).

## Description

Many of the 131 cells that die during *Caenorhabditis elegans* development are the smaller daughter of a neuroblast (referred to as ‘mother' cell) that divides asymmetrically by both fate and size (Sulston and Horvitz, 1977; Sulston et al., 1983). Loss-of-function mutations of *pig-1* , which encodes a PAR-1-like kinase orthologous to human MELK (maternal embryonic leucine zipper kinase), cause mother cells to divide symmetrically by size, thereby increasing the physical size of the normally smaller daughter cell i.e. the cell programmed to die (Cordes et al., 2006). In addition, these mutations cause inappropriate survival of cells programmed to die, indicating that they compromise the ability of these cells to undergo apoptosis (Cordes et al., 2006). Conversely, gain-of-function mutations of *ect-2* , which encodes the *C. elegans* ortholog of the human RhoGEF Ect2 (Epithelial Cell Transforming 2), cause mother cells to divide more asymmetrically by size, thereby decreasing the physical size of the already smaller daughter cell i.e. the cell programmed to die (Sethi et al., 2022). In addition, these *ect-2* gain-of-function mutations partially suppress the inappropriate survival of cells programmed to die caused by partial loss-of-function mutations of *ced-3* (which encodes a caspase required for apoptosis in *C. elegans* ), indicating that they promote the ability of these cells to undergo apoptosis (Sethi et al., 2022). However, at least in the NSM neuroblast lineage ( **Figure 1** ; see below), mutations in both *pig-1* and *ect-2* also affect the unequal inheritance of cellular components during asymmetric mother cell division (Wei et al., 2020; Sethi et al., 2022). For instance, during asymmetric NSM neuroblast division, the loss of *pig-1* disrupts the unequal segregation - into the surviving NSM – of the anti-apoptotic transcription factor CES-1 , an ortholog of human SCRT1 and 2 (scratch family transcriptional repressor). As a result, the NSM sister cell, which is programmed to die, now inherits this anti-apoptotic factor (Wei et al., 2020). For this reason, it has been difficult to discern whether the inappropriate survival of cells programmed to die observed in *pig-1* loss-of-function mutants is the result of their increased size, the inappropriate inheritance of anti-apoptotic factors or both. The rationale for the study described below was to change the physical size of cells programmed to die using genetic tools other than mutations that affect the ability of mother cells to divide asymmetrically and to determine the impact of this change on the ability of the resulting cells to undergo apoptosis.

The *C. elegans* genes *ima-3* and *C27D9.1* encode an importin alpha protein and a protein predicted to be involved in fucosylation, respectively (Geles and Adams, 2001; WormBase). The knock-down by RNA interference (RNAi) of *ima-3* and *C27D9.1* decreases or increases embryo size, respectively (Sönnichsen et al., 2005; Hara and Kimura, 2009; Weber and Brangwynne, 2015; Hubatsch et al., 2019). Using *sorb-1 (RNAi)* as a control (Haeussler et al., 2021), we confirmed this in wild type and in different *ced-3* caspase mutant backgrounds, in which apoptosis is compromised with increasing degrees i.e. *ced-3 ( n2438 )* , *ced-3 ( n2427 )* , *ced-3 ( n2436 )* and *ced-3 ( n717 )* ( **Figure 1A, B** ; see below) (Ellis and Horvitz, 1986; Shaham et al., 1999). In addition, by measuring the size of the NSM neuroblast just prior to its division (metaphase; performed in the *ced-3 ( n2427 )* background), we found that *ima-3 (RNAi)* reduces NSM neuroblast size whereas *C27D9.1 (RNAi)* increases NSM neuroblast size albeit not significantly ( **Figure 1C, D** ). The two NSM neuroblasts divide and each gives rise to a larger NSM, which survives, and a smaller NSM sister cell (NSMsc), which is programmed to die through apoptosis ( **Figure 1E** ). Next, using a reporter that visualizes the two NSMs as well as inappropriately surviving NSM sister cells in L4 larvae (P *_tph-1_ his-24 ::gfp* ; transgene *bcIs66* ) (Yan et al., 2013) ( **Figure 1E** ), we determined ‘NSM sister cell survival' (% NSMsc survival) in *ima-3 (RNAi)* and *C27D9.1 (RNAi)* larvae in a wild-type background (+/+) and in the background of *ced-3 ( n2438 )* , *ced-3 ( n2427 )* , *ced-3 ( n2436 )* or *ced-3 ( n717 )* , respectively. In a wild-type background, NSM sister cells always die (0% NSMsc survival), and we found that *ima-3* ( *RNAi* ) has no effect on this ( **Figure 1F,** *sorb-1 (RNAi)* vs *ima-3 (RNAi)* ). In the background of the weak *ced-3* loss-of-function mutations *ced-3 ( n2438 )* and *ced-3 ( n2427 )* , *ima-3 (RNAi)* suppresses NSM sister cell survival from 10% to 6% and from 12% to 7%, respectively. Therefore, in these mutant backgrounds, *ima-3 (RNAi)* causes increased NSM sister cell death, which indicates that *ima-3* has ‘anti-apoptotic' function. However, *ima-3 (RNAi)* has no effect on NSM sister cell survival in the background of the stronger *ced-3* loss-of-function mutation *ced-3 ( n2436 )* or the putative *ced-3* null mutation *ced-3 ( n717 )* , in which 65% or 100% of the NSM sister cells inappropriately survive ( **Figure 1F** ). In the wild-type background, *C27D9.1* ( *RNAi* ) also has no effect on NSM sister cell survival ( **Figure 1F,** *sorb-1* ( *RNAi* ) vs *C27D9.1* ( *RNAi* )). However, we found that *C27D9.1 (RNAi)* enhances NSM sister cell survival in *ced-3 ( n2438 )* , *ced-3 ( n2427 )* and *ced-3 ( n2436 )* animals but not in *ced-3 ( n717 )* animals, in which NSM sister cell survival is already 100% ( **Figure 1F** ). Therefore, in these mutant backgrounds, *C27D9.1 (RNAi)* causes decreased NSM sister cell death, which indicates that *C27D9.1* has ‘pro-apoptotic' function.

At least to our knowledge, *ima-3* and *C27D9.1* have not been implicated in apoptosis or non-apoptotic forms of cell death. While we cannot rule out that they have not yet described roles in cell death, the simplest explanation for our results is that *ima-3* is anti-apoptotic and *C27D9.1* pro-apoptotic as a result of their opposing roles in embryo size and NSM neuroblast size and therefore, presumably, the size of the NSM sister cell. Specifically, we propose that – at least in the background of partial loss-of-function mutations of *ced-3* caspase - the *ima-3 (RNAi)* -induced reduction in cell size promotes and the *C27D9.1 (RNAi)* -induced increase in cell size compromises the ability of the NSM sister cell to undergo apoptosis. Hence, our results support the notion that in *C. elegans* embryos, cell size and apoptosis inversely correlate. Our results also suggest that the effects of *pig-1* and *ect-2* mutations on the ability of the NSM sister cell to undergo apoptosis are in part due to their impact on NSM sister cell size. Finally, our finding that in a wild-type background, *ima-3 (RNAi)* and *C27D9.1 (RNAi)* do not affect cell death in the NSM neuroblast lineage suggests that the *ima-3 (RNAi)* -induced reduction in cell size is not sufficient to cause inappropriate apoptosis (of the NSM neuroblast or the NSM) and that the *C27D9.1 (RNAi)* -induced increase in cell size is not sufficient to cause the NSM to inappropriately survive; however, we cannot exclude that the *ima-3 (RNAi)* -induced reduction in cell size and the *C27D9.1 (RNAi)* -induced increase in cell size were not large enough to impact apoptosis.

## Methods

**Strain maintenance and genetics**

Strains were maintained at 15°C or 20°C on Nematode Growth Media (NGM) plates and fed with OP50 *Escherichia coli* bacteria according to standard protocols (Brenner, 1974). Bristol N2 strain was used as wild type. The following transgenes and alleles were used in this study: LGIII *bcIs66* ( *P _tph-1_ his-24 ::gfp* ) (Yan et al., 2013); LGIV *ced-3 ( n2438 ), ced-3 ( n2427 ), ced-3 ( n2436 ), ced-3 ( n717 )* (Ellis and Horvitz, 1986; Shaham et al., 1999); LGV *ltIs44* ( *P _pie-1_ mCherry:: ph ^PLCΔ^* ) (Audhya et al., 2005). LGII *bcIs104* ( *P _pie1_ gfp:: tac-1* ) (Bellanger and Gonczy, 2003). RNAi clones from the Ahringer RNAi library were used for the following genes (Kamath and Ahringer, 2003; Kamath et al., 2003): *ima-3* ( *F32E10.4* ) (Sönnichsen et al., 2005), *C27D9.1* (Sönnichsen et al., 2005), *sorb-1* ( *Y45F10D.13* ) (Haeussler et al., 2021), and *pop-1* ( *W10C8.2* ) (Rueyling et al., 1998). Throughout our studies, we used information and tools available on WormBase ( https://wormbase.org/#012-34-5 ) (Fire et al., 1998; Sternberg et al., 2024).

**RNA interference** ( **RNAi) by feeding**

All RNAi bacterial clones were picked from the Ahringer library (Kamath and Ahringer, 2003; Kamath et al., 2003). This library is distributed by Source BioScience Ltd. ( https://sourcebioscience.com ). To verify the clones, genomic fragments of the targeted genes cloned into the L4440 vector were amplified by PCR using L4440-F ( TGGATAACCGTATTACCGCC ) and L4440-R ( GTTTTCCCAGTCACGACGTT ) oligos, sequenced and sequences verified using the WormBase BLAST tool ( https://wormbase.org/tools/blast_blat ). Verified RNAi bacterial clones were inoculated in 200 µL of LB broth containing 100 μg/mL carbenicillin and grown overnight at 37°C in a shaking incubator. 100 µL of each bacterial culture was seeded onto NGM plates containing 6 mM IPTG and 100 µg/mL carbenicillin. Plates were kept at room temperature overnight in the dark. RNAi by feeding was performed by transferring L4 larvae onto seeded IPTG plates and kept at 20°C for 24-96 hours depending on the gene (Timmons and Fire, 1998; Burton et al., 2011). F1 embryos and/or L4 larvae were harvested for embryo or cell size analyses and for determining NSM sister cell survival. *sorb-1 (RNAi)* was used as negative control (Haeussler et al., 2021) and *pop-1 (RNAi)* as positive control (Rueyling et al., 1998) for RNAi. Loss of *pop-1* resulted in nearly 100% dead embryos.

**Microscopy**

Measurements of embryo length and NSM neuroblast (NSMnb) size

24-48 hours post-RNAi feeding, embryos were harvested for embryo size measurements. Embryos were mounted on a glass slide with 2% agarose pads containing M9 buffer. For measuring the lengths of the embryos, Nomarski Z-stacks were taken. The central Z-stack was used to measure the length of each embryo. Using a line selection tool in ImageJ software, a straight line was drawn in the centre of the embryo from anterior to posterior (egg shell), and the length was measured by using ImageJ software (https://imagej.net/ij/) (Schneider et al., 2012; v1.54m). Average embryo length was calculated for each RNAi condition.

The size of the NSMnb under different RNAi conditions was estimated using the *P _pie-1_ mCherry::ph ^PLCΔ^* plasma membrane reporter ( *ltIs44* ) (Audhya et al., 2005) essentially as described (Sethi et al., 2022). The NSMnb was identified based on its morphology and position and its size was estimated when it had reached metaphase. A Z-stack (0.5 μm step size) was taken through it, and a region of interest (ROI) was drawn around the plasma membrane of the NSMnb in all Z-slices to determine NSMnb area. To estimate NSMnb size, the sum was taken of all NSMnb areas.

A Zeiss Axio Imager.M2 equipped with Differential Interference Contrast (DIC) and epifluorescence was used for imaging.

Measurement of % NSM sister cell (NSMsc) survival

72-96 hours post-RNAi feeding start, L3/L4 larvae were picked to score inappropriate NSMsc survival using the *P _tph-1_ his-24 ::gfp* reporter ( *bcIs66* ). Worms were mounted on a glass slide with 2% agarose pads containing sodium azide in M9 buffer (25 mM). NSMsc survival was determined by counting GFP-positive cells in the anterior pharynx as previously described (Thellmann et al., 2003; Yan et al., 2013; Sethi et al., 2022). A Zeiss Axio Imager.M2 equipped with Differential Interference Contrast (DIC) and epifluorescence was used for imaging.

**Statistical analyses**

Statistical analyses were performed using the R statistical software package. For comparing the embryo sizes, a linear model containing all genotypes and RNAi conditions was constructed (R Core Team, 2024; v4.4.1), and its residuals were visually inspected for deviations from normality and homoscedasticity of variance. Differences between relevant RNAi treatments ( *sorb-1 (RNAi)* controls vs respective RNAi treatments) within genotypes were then tested post-hoc using the least square means method in the *emmeans* package (Lenth, 2024; v1.10.5). To compare statistical significance of NSMnb size under different RNAi conditions, Mann-Whitney U-tests were used. One sided Fisher's exact tests were used to determine statistical significance of % NSMsc survival under all RNAi conditions. All *p-values* were corrected for multiple testing using false discovery rate. (Benjamini and Hochberg, 1995). Significance level (α) was set at *p* < 0.05 for all tests.

## Reagents

**Table:** 

Strain	Genotype	Source
MD2487	*bcIs66 III*	Yan et al., 2013
MD4972	* bcIs66 III; ced-3 ( n2438 ) IV *	This study
MD3712	* bcIs66 III; ced-3 ( n2427 ) IV; ltIs44 V *	Chakraborty et al., 2015
MD3588	* bcIs66 III; ced-3 ( n2436 ) IV; ltIs44 V; bcIs104 *	Chakraborty et al., 2015
MD3203	* bcIs66 III; ced-3 ( n717 ) IV *	Yan et al., 2013

Complete genotypes of strains used in this study.
